# Common variations in TERT-CLPTM1L locus are reproducibly associated with the risk of nasopharyngeal carcinoma in Chinese populations

**DOI:** 10.18632/oncotarget.6397

**Published:** 2015-11-26

**Authors:** Yang Zhang, Xiaoai Zhang, Hongxing Zhang, Yun Zhai, Zhifu Wang, Peiyao Li, Lixia Yu, Xia Xia, Ying Zhang, Yixin Zeng, Fuchu He, Gangqiao Zhou

**Affiliations:** ^1^ State Key Laboratory of Proteomics, Beijing Proteome Research Center, Beijing Institute of Radiation Medicine, Beijing, China; ^2^ Institute of Basic Medical Sciences, Chinese Academy of Medical Sciences and Peking Union Medical College, Beijing, China; ^3^ State Key Laboratory of Pathogen and Biosecurity, Beijing Institute of Microbiology and Epidemiology, Beijing, China; ^4^ Department of Experimental Research, Sun Yat-sen University Cancer Center, Guangzhou, China

**Keywords:** TERT, CLPTM1L, polymorphism, nasopharyngeal carcinoma

## Abstract

Associations between single nucleotide polymorphisms (SNPs) at 5p15 (*TERT-CLPTM1L*) and multiple cancer types have been reported. We examined whether polymorphisms in the *TERT-CLPTM1L* locus were related to the risk of developing nasopharyngeal carcinoma (NPC) among Chinese populations. In the first stage, 26 tag SNPs were genotyped in a Guangxi population (855 patients and 1036 controls). In the second stage, the SNPs, which showed significant association, were further genotyped in a Guangdong population (997 patients and 972 controls). Functional analyses were conducted to verify the biological relevance of the associated polymorphism. In the 1st stage, four SNPs (rs2736098, rs2735845, rs402710, and rs401681) were significantly associated with the risk of developing NPC. After the 2nd stage validation, rs2735845 and rs401681 were independently associated with the risk of developing NPC in the additive model (rs2735845, OR = 1.19, 95% CI = 1.04–1.37, *P* = 0.011; rs401681, OR = 0.85, 95% CI = 0.74–0.99, *P* = 0.034). Furthermore, we observed higher *CLPTM1L* messenger RNA levels in fetal mesenchymal stem cells from the rs2735845 *G* allele carriers compared with that from non-carriers. In addition, using an immunohistochemistry assay, we observed higher TERT and CLPTM1L levels in NPC tissues compared with that in non-cancerous nasopharyngeal tissues. Our findings suggest that polymorphisms in the *TERT-CLPTM1L* locus may play a role in mediating the susceptibility to NPC in Chinese populations.

## INTRODUCTION

Nasopharyngeal carcinoma (NPC) is an epithelial malignancy of the head and neck. The incidence of NPC is higher in Southeast Asia and Africa but lower among Caucasians in North America and Europe. In southern China, the incidence rate is approximately 15–50 per 100,000 person-years, which is 100-fold higher than that in the Western world [1]. NPC is a complex disease caused by an interaction among Epstein-Barr virus infection, environmental risk factors and host genes in a multi-step process of carcinogenesis [2]. The identification of susceptibility genes contributing to NPC may help clarify the pathogenesis of carcinogenesis and improve the prevention and treatment of this malignancy.

The human 5p15.33 locus contains two well-known genes, the telomerase reverse transcriptase (*TERT*) and the cleft lip and palate transmembrane 1-like (*CLPTM1L*) genes, which have been implicated in carcinogenesis. TERT, as the rate-limiting catalytic subunit of the telomerase enzyme, plays an essential role in the maintenance of telomere DNA length, chromosomal stability, and cellular immortality [3]. TERT is expressed in the vast majority of human malignant celllines and tumors but not in the corresponding benign tissues [4]. With regard to NPC, elevated telomerase activity and TERT expression levels were detected in tumor tissues compared with normal nasopharyngeal epithelium and tissues [5, 6]. Furthermore, for the clinicopathological features in NPC patients, the telomerase activity was observed more frequently in advanced clinical stage and lymph node metastasis [7, 8]. The other gene in this region, *CLPTM1L*, which is named for its similarity to a gene implicated in the susceptibility to cleft lip palate, was identified through screening for cisplatin-resistance-related genes. Although the function of this gene is less known, it was found to be upregulated in cisplatin-resistant ovarian tumor cell lines and to induce apoptosis in cisplatin-sensitive cells [9]. On the basis of the above functional relevance of the *TERT* and *CLPTM1L* genes in the pathogenesis of cancers, we hypothesize that *TERT* and *CLPTM1L* may be excellent biological candidate susceptibility genes for cancers.

Single nucleotide polymorphisms (SNPs) in the *TERT*-*CLPTM1L* locus on chromosome 5p15.33 have been linked to a spectrum of cancers. Multiple independent genome-wide association studies (GWAS) have shown that SNPs in the *TERT*-*CLPTM1L* locus were significantly associated with the risk of many different types of cancer, including basal cell carcinoma, glioma, lung, cervical, prostate, bladder, pancreatic, chronic lymphocytic leukemia, and testicular germ cell cancers [10–17]. Association studies on candidate gene strategy also reported that polymorphisms in the *TERT*-*CLPTM1L* locus were associated with serous ovarian, breast, endometrial, lung, and colorectal cancers [18–20]. The roles of the polymorphism in the *TERT*-*CLPTM1L* locus in NPC, however, have not been fully investigated. In the present study, we examined whether the polymorphism in the *TERT*-*CLPTM1L* locus have any bearing on the risk or severity of NPC in Chinese populations.

## RESULTS

### Genetic association between polymorphisms in the *TERT-CLPTM1L* locus and NPC

Among the 26 tag SNPs chosen first, four SNPs were eliminated because of low frequency (rs2853691), poor genotyping reliability (rs2736118), or departure from Hardy-Weinberg equilibrium (*P* < 0.05) (rs2853668 and rs10073340). We therefore selected the remaining 22 SNPs for the subsequent analyses.

**Figure 1:**
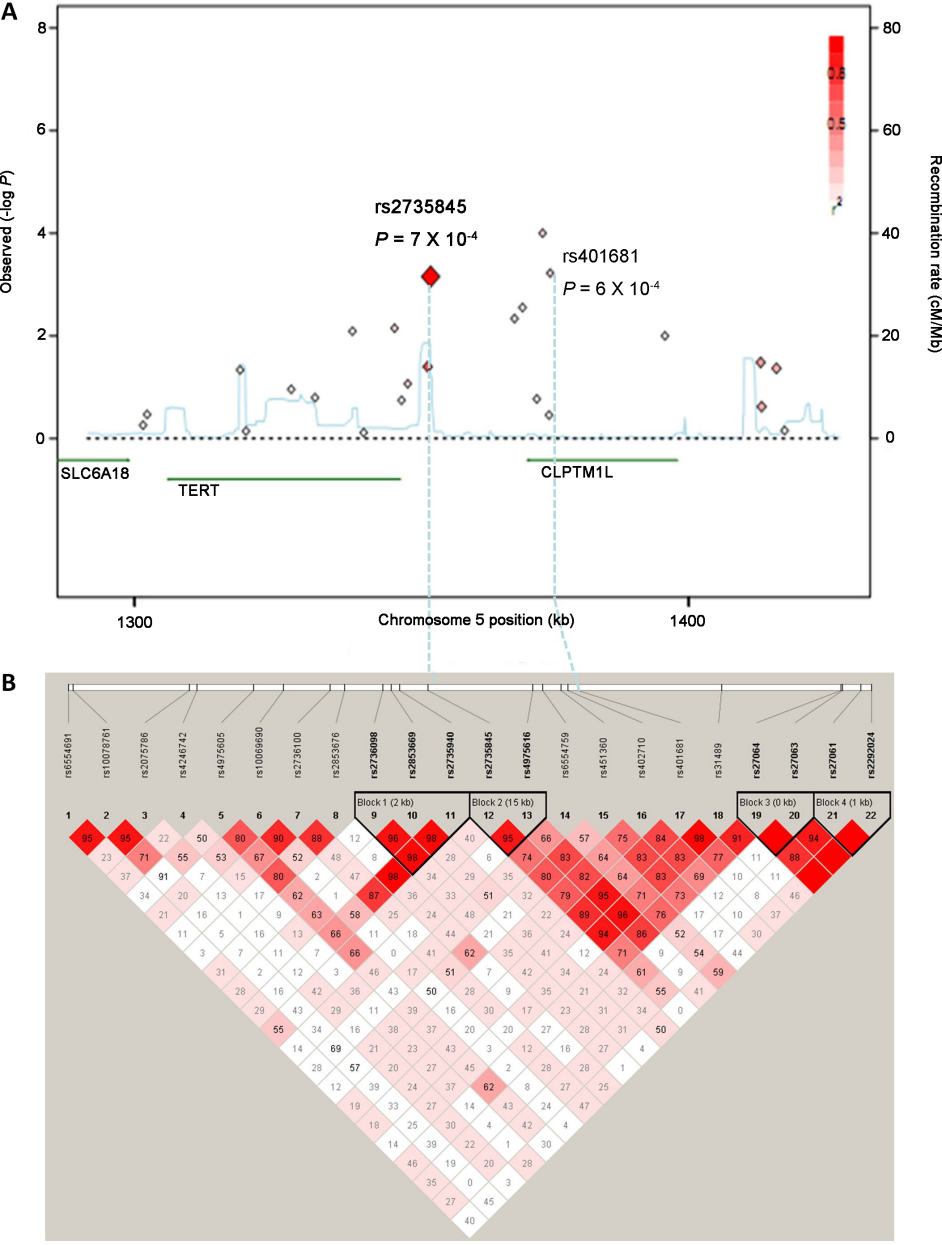
Regional plots for associations at the *TERT-CLPTM1L* locus on chromosome 5p15.33 (**A**) Significance of the each tag SNPs. Shown are the −log_10_ association *P* values of SNPs in the Guangxi population. The intensity of the red shading indicates the strength of the linkage disequilibrium (LD) with the index SNP (rs2735845). Also shown are the SNP built 36 coordinates in kilobases (kb), the recombination rates in centimorgans (cM) per megabase (Mb) (in blue) and the genes in the region (in green). (**B**) Pairwise LD between tag SNPs at the *TERT-CLPTM1L* locus. The value within each diamond represents the pairwise correlation between tag SNPs (measured as *D*’) defined by the upper left and the upper right sides of the diamond. The diamond without a number corresponds to *D*’ = 1. Shading represents the magnitude and significance of pairwise LD, with a red-to-white gradient reflecting higher to lower LD values.

The genotyping results for the 22 SNPs are shown in Table 1 and Figure 1. The observed genotype frequencies for the 22 polymorphisms conformed to Hardy–Weinberg equilibrium in the Guangxi population (all *P* > 0.05, data not shown). On the basis of logistic regression analysis with adjustment for age, sex, smoking status, alcohol use, and family history, significant associations with the susceptibility to NPC were observed with the 12 SNPs in the Guangxi population (Table 1). After multiple corrections, four SNPs (rs2736098, rs2735845, rs402710, and rs401681) were significantly associated with NPC risk (*P* < 0.0029 after SNP SpD correction, Meff Li value = 17, Table 1). We also investigated the association between the 22 SNPs and the severity of NPC (as measured by TNM staging system) in the Guangxi population. After multiple corrections, no association was observed between the 22 SNPs and the severity of NPC (Table S8).

**Table 1:** Associations between tag SNPs on chromosome 5p15.33 and risk of nasopharyngeal carcinoma in the Guangxi population

Genes	SNPs	Chromo-some position	Major allele / Minor allele	Major allele frequency	Genotypes			OR (95% CI)^b^	*P* value^b^
					Common^a^	Heterozy-gous^a^	Rare^a^		
	rs6554691	1301873	T/A	0.210	65.1/64.3	31.1/31.2	3.8/4.5	0.96 (0.82–1.14)	0.65
	rs10078761	1302594	T/A	0.033	94.5/93.5	5.3/6.3	0.1/0.2	0.84 (0.57–1.23)	0.36
*TERT*	rs2075786	1319310	A/G	0.208	67.4/63.0	28.7/38.4	4.0/4.6	0.84 (0.71–0.99)	0.037
*TERT*	rs4246742	1320356	T/A	0.455	30.7/29.8	48.7/49.3	20.6/20.9	0.97 (0.85–1.11)	0.68
*TERT*	rs4975605	1328528	C/A	0.159	74.9/70.7	21.8/26.9	3.3/2.4	0.85 (0.71–1.02)	0.073
*TERT*	rs10069690	1332790	C/T	0.130	72.6/75.6	25.9/23.0	1.5/1.4	1.16 (0.96–1.41)	0.13
*TERT*	rs2736100	1339516	T/G	0.399	31/35.2	50/49.8	19/15	1.21 (1.06–1.39)	0.0055
*TERT*	rs2853676	1341547	C/T	0.144	74.7/73.4	22.5/24.4	2.9/2.2	0.97 (0.80–1.17)	0.74
*TERT*	rs2736098	1347086	C/T	0.398	29.9/37.8	51.9/44.9	18.2/17.3	1.27 (1.09–1.46)	0.0014*
*TERT*	rs2853669	1348349	C/T	0.365	38.2/41.7	46.2/43.5	15.6/14.8	1.10 (0.96–1.26)	0.16
*TERT*	rs2735940	1349486	A/G	0.498	28.1/25.2	49.3/49.1	22.6/25.6	0.89 (0.78–1.01)	0.072
	rs2735845	1353584	C/G	0.361	34.3/41.7	47.7/44.4	17.9/13.9	1.26 (1.10–1.44)	0.00070*
	rs4975616	1368660	A/G	0.261	59.8/53.6	35.8/40.5	4.5/5.8	0.79 (0.68–0.93)	0.0038
*CLPTM1L*	rs6554759	1370102	A/G	0.100	86.8/81.9	13.0/17.4	0.2/0.7	0.69 (0.54–0.89)	0.0038
*CLPTM1L*	rs451360	1372680	T/G	0.186	69.2/66.8	27.9/29.3	2.9/3.9	0.87 (0.73–1.03)	0.11
*CLPTM1L*	rs402710	1373722	C/T	0.350	49.8/42.0	42.3/46.3	7.9/11.7	0.76 (0.66–0.87)	0.00010*
*CLPTM1L*	rs401681	1375087	C/T	0.347	49.0/42.9	43.2/44.8	7.8/12.3	0.78 (0.68–0.90)	0.00060*
*CLPTM1L*	rs31489	1395714	C/A	0.257	60.4/54.4	34.8/39.7	4.9/5.9	0.81 (0.69–0.95)	0.0087
	rs27064	1412938	C/T	0.296	45.3/50.8	43.4/39.2	11.3/10.0	1.17 (1.02–1.35)	0.027
	rs27063	1413125	G/C	0.488	23.9/26.6	50.4/49.3	25.7/24.1	1.09 (0.95–1.24)	0.22
	rs27061	1415793	C/T	0.483	23.8/26.9	48.6/49.7	27.6/23.5	1.15 (1.01–1.31)	0.034
	rs2292024	1417242	C/G	0.126	76.7/76.5	20.5/21.9	2.8/1.6	1.04 (0.86–1.26)	0.69

Abbreviations: OR, odds ratio; CI, confidence interval.

a Frequency of the genotype in the patients/frequency of the genotype in the controls.

b The ORs and *P* values were adjusted for age, sex, smoking and drinking status, smoking level, nationality, and family history of cancer in the additive model.

* The *P* value remained significant after correction for multiple comparisons.

To confirm the initial associations of these four SNPs with NPC, the Guangdong population, composed of 997 patients and 972 controls, was also genotyped. The observed genotype frequencies for the four polymorphisms conformed to Hardy-Weinberg equilibrium in the Guangdong population (all *P* > 0.05, data not shown). There was no association between the rs2736098 and rs402710 polymorphisms and the risk of NPC in the Guangdong population (data not shown). However, the rs2735845 and rs401681 polymorphisms were significantly associated with risk of NPC in the Guangdong population (Table 2). For the 2735845 polymorphism, the *G* allele presented significant association with increased risk of NPC in the additive model (OR = 1.19, 95% CI = 1.04–1.37, *P* = 0.011). For the rs401681 polymorphism, the *T* allele presented significant association with decreased risk of NPC in the additive model (OR = 0.85, 95% CI = 0.74–0.99, *P* = 0.034). When the Guangxi and Guangdong populations were combined, stronger associations were observed between both the rs2735845 and rs401681 polymorphisms and NPC risk in the additive model (for rs2735845: adjusted OR = 1.23, 95% CI = 1.12–1.35, *P* = 4.64 × 10^−5^; for rs401681: adjusted OR = 0.81, 95% CI = 0.74–0.90, *P* = 1.00 × 10^−4^) (Table 2). The effects of rs2735845 and rs401681 were maintained when corrected for each other by logistic regression (OR = 1.15, 95% CI = 1.03–1.29, *P* = 0.012 and OR = 0.88, 95% CI = 0.78–0.99, *P* = 0.03, respectively). No association more significant than that of rs2735845 and rs401681 was identified through imputation of other polymorphisms in the *TERT-CLPTM1L* locus based on HapMap CHB data (data not shown). Furthermore, the correlation between rs2735845 and rs401681 is not strong (*D*’ = 0.91, *r*^2^ = 0.24 in HapMap CHB samples, *D*’ = 0.89, *r*^2^ = 0.24 in our control data; Figure 1). Taken together, these data strongly suggest that rs2735845 and rs401681 are independently associated with NPC.

**Table 2:** The genotype frequencies of rs2735845 and rs401681 polymorphisms in patients and controls of the Guangxi and Guangdong population

SNPs and genotypes	Guangxi population			Guangdong population			Combined		
	Frequency^a^	OR (95% CI)^b^	*P* value^b^	Frequency^a^	OR (95% CI)^b^	*P* value^b^	Frequency^a^	OR (95% CI)^c^	*P* value^b^
rs2735845									
CC	34.3/41.7	Reference		32.1/38.8	Reference		33.0/40.4	Reference	
GC	47.7/44.4	1.33 (1.08–1.63)	0.007	51.9/45.4	1.37 (1.12–1.69)	0.002	50.0/45.0	1.36 (1.18–1.57)	2.00 × 10^−5^
GG	17.9/13.9	1.58 (1.19–2.09)	0.001	16.0/15.8	1.30 (0.98–1.73)	0.058	17.0/14.7	1.42 (1.16–1.73)	3.90 × 10^−4^
Recessive model		1.35 (1.05–1.75)	0.022		1.08 (0.84–1.40)	0.54		1.19 (1.00–1.43)	0.049
Additive model		1.26 (1.10–1.44)	7.00 × 10^−4^		1.19 (1.04–1.37)	0.011		1.23 (1.12–1.35)	4.64 × 10^−5^
rs401681									
CC	49.0/42.9	Reference		50.3/46.1	Reference		49.7/44.5	Reference	
CT	43.2/44.8	0.84 (0.69–1.02)	0.08	42.2/43.6	0.91 (0.75–1.11)	0.28	42.6/44.2	0.85 (0.73–0.98)	0.032
TT	7.8/12.3	0.55 (0.39–0.77)	4.50 × 10^−4^	7.5/10.2	0.68 (0.48–0.97)	0.028	7.6/11.3	0.57 (0.44–0.73)	1.90 × 10^−5^
Recessive model		0.60 (0.43–0.82)	0.001		0.71 (0.51–1.00)	0.048		0.61 (0.48–0.78)	1.20 × 10^−4^
Additive model		0.78 (0.68–0.90)	6.00 × 10^−4^		0.85 (0.74–0.99)	0.034		0.81 (0.74–0.90)	1.00 × 10^−4^

a Frequency of the genotype in the patients/frequency of the c genotype in the controls.

b The ORs and *P* values were calculated by logistic regression analysis adjusted for age, sex, smoking and drinking status, smoking level, nationality (only in Guangxi population), and family history of cancer.

The associations between the rs2735845 and rs401681 polymorphisms and the susceptibility to NPC in both the Guangxi and Guangdong populations were further examined with stratification by age, sex, smoking status, smoking level, drinking status and family of history. For the rs401681 polymorphism, although the susceptibility to NPC seemed to be more pronounced in some subgroups, these differences could be attributed to chance (all *P* > 0.05, test for homogeneity; data not shown). For the rs2735845 polymorphism, the general pattern of results was similar (data not shown).

### Effects of the rs2735845 and rs401681 polymorphisms on *TERT* and *CLPTM1L* mRNA expression

**Figure 2:**
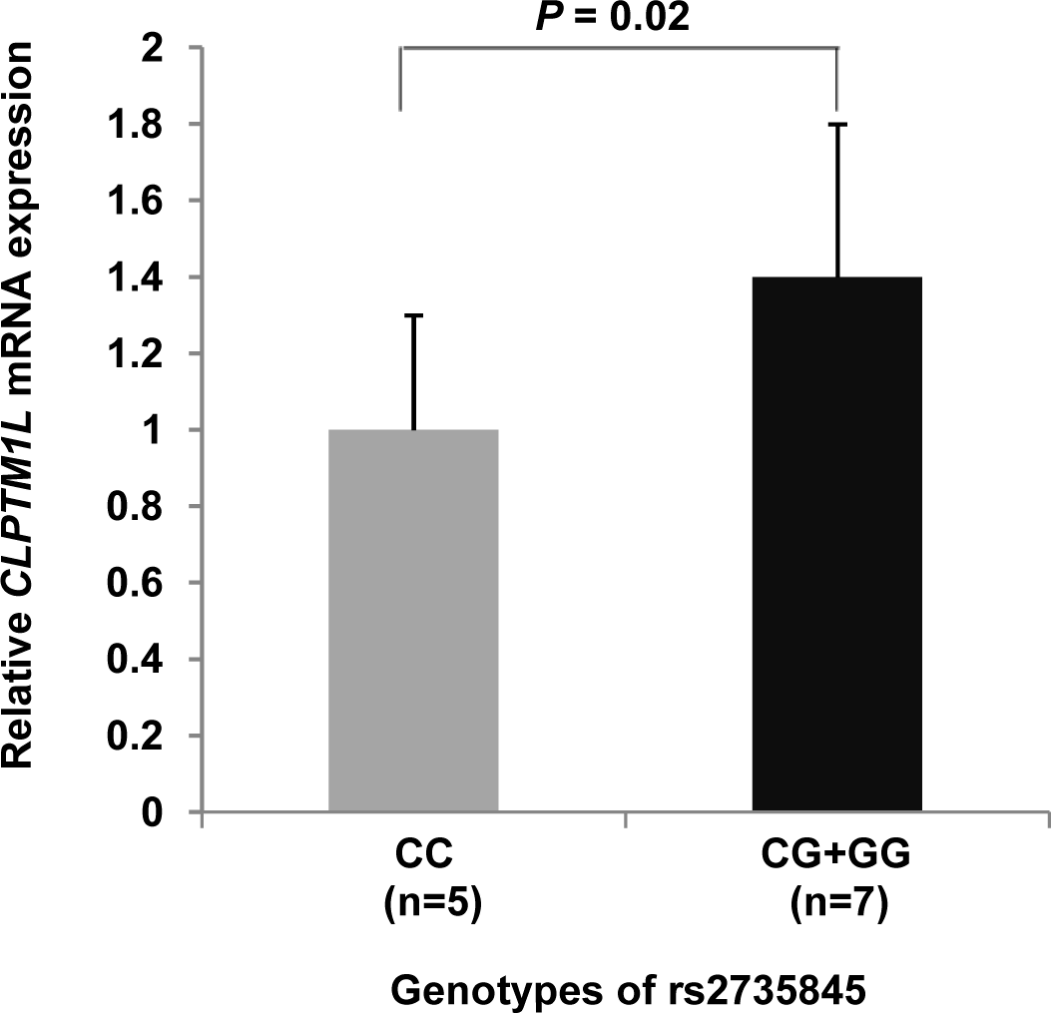
Quantitative real-time RT-PCR for *CLPTM1L* The expression of *CLPTM1L* for three different genotypes of rs2735845 was measured in RNA from fetal mesenchymal stem cells from human umbilical cord obtained from a group of 12 normal full-term-delivery women. Normalization for mRNA quantity was performed with human *GAPDH* control primers for each sample, and the final abundance values were adjusted to yield an arbitrary value of 1 for the rs2735845 CC genotype. Columns include the mean from triplicate measurements; the vertical bars indicate the standard deviation. Compared to the CC carriers, the *G* allele carriers exhibited markedly elevated *CLPTM1L* transcription (*P* = 0.02; *t* test).

We evaluated the association between genotypes of the rs2735845 and rs401681 polymorphisms and *TERT* and *CLPTM1L* mRNA levels in 12 fetal mesenchymal stem cells (fMSCs). As shown in Figure 2, the relative *CLPTM1L* mRNA level from fMSCs with rs2735845 *G* allele (GG + CG genotypes) was significantly higher than that from fMSCs with CC genotype (*t* test; *P* = 0.02). However, there was no association between *CLPTM1L* mRNA level and rs401681 genotypes in fMSCs (data not shown).

### Greater TERT and CLPTM1L expression in NPC tissues compared with that in non-cancerous nasopharyngeal tissues

**Figure 3:**
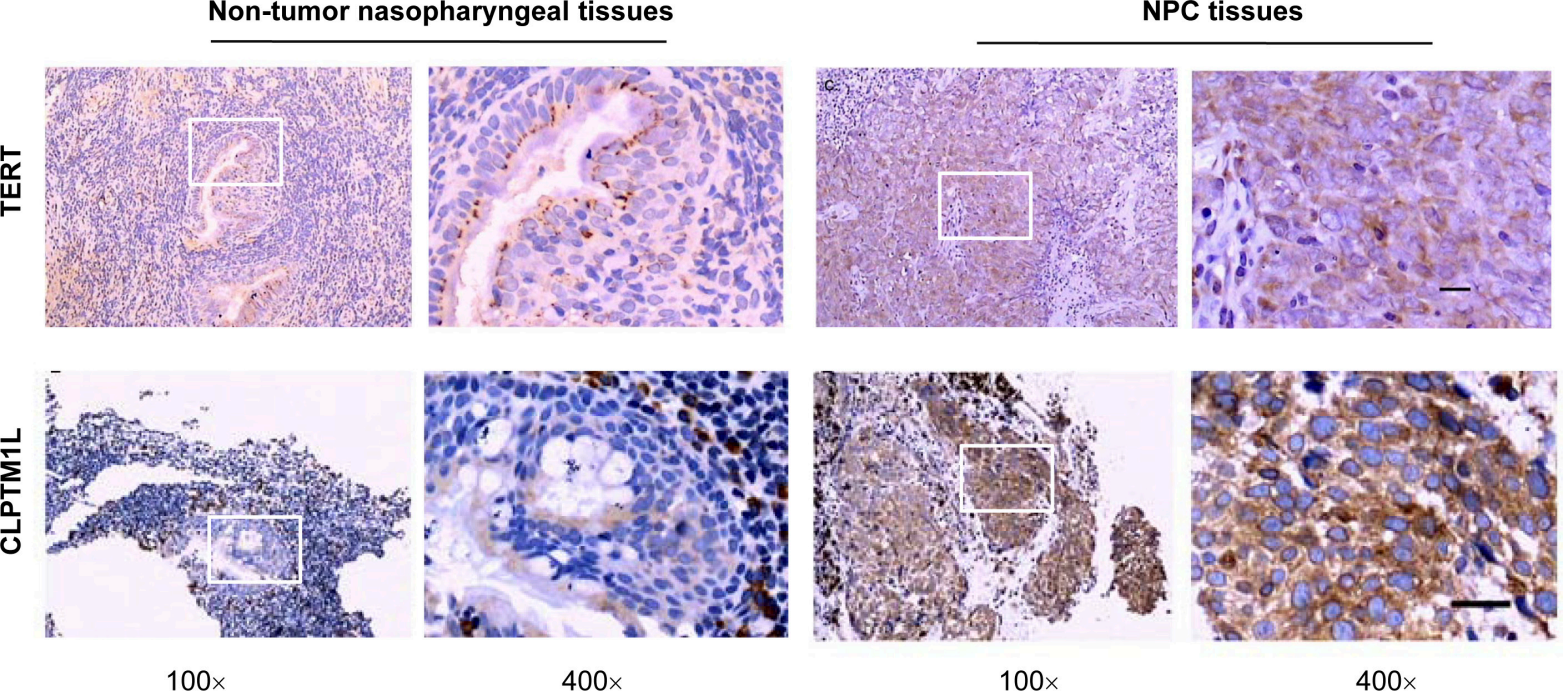
Protein expression of TERT and CLPTM1L by immunohistochemical staining in representative nasopharyngeal carcinoma tissues and non-tumor nasopharyngeal tissues Images in the box (left, 100×) were enlarged and are shown in the right box (400×). The scale bar represents 50 μm.

We investigated the protein expression of TERT and CLPTM1L in NPC tissues and non-cancerous nasopharyngeal tissues by immunohistochemical (IHC) assay (Figure 3 and Table S1 in Supplementary Materials). Significantly higher expression of TERT was observed in the NPC tissues compared to that in the non-cancer nasopharyngeal tissues (*P* < 0.001). There was also significantly higher expression of CLPTM1L in the NPC tissues compared to that in the non-cancer nasopharyngeal tissues (*P* < 0.001). However, there was no association between the genotypes of the rs2735845 and rs401681 polymorphisms and the expression of TERT and CLPTM1L in NPC tissues and non-cancerous nasopharyngeal tissues.

## DISCUSSION

In this study, we investigated the associations of 26 tag SNPs in the *TERT*-*CLPTM1L* locus on 5p15.33 with the risk of occurrence and progression of NPC in populations in southern China. We found that two SNPs, rs2735845 and rs401681, were significantly associated with the onset of NPC in two independent populations. Functional analysis showed that the rs2735845 *G* allele was associated with higher *CLPTM1L* mRNA levels when compared with the *C* allele. In addition, we also found that the expression of TERT and CLPTM1L was higher in the NPC tissues compared to the non-cancer nasopharyngeal tissues. Our data, together with recent evidence for an association of the polymorphisms in the *TERT*-*CLPTM1L* locus with the risk of lung and colorectal cancer [19, 20], suggest that the polymorphisms at this locus are potent genetic risk factors for the onset of NPC.

The genetic associations observed in this study are biologically plausible. There are two genes within the 5p15.33 *TERT*-*CLPTM1L* locus through which the association might plausibly be mediated: *TERT* and *CLPTM1L*. *TERT* translates the catalytic protein subunit of the telomerase complex, which elongates the telomere in cells. Telomere-driven genome instability is a widespread cause of genome instability in cancer and is thought to be the crucial event in the development of tumorigenesis [3]. It has been shown that small hairpin RNA can direct against TERT to inhibit cell viability by regulating telomerase activity and its related protein expression in NPC cells [21]. Moreover, overexpression of TERT has been detected in a variety of cancer cells, including NPC [6]. In this study, we also found that the expression of TERT was higher in the NPC tissues compared to the non-cancer nasopharyngeal tissues. Our results, together with those of the earlier studies, suggest that TERT plays an important role in NPC carcinogenesis. CLPTM1L encodes a predicted transmembrane protein, which plays an important role in carcinogenesis. CLPTM1L is upregulated in a cisplatin-resistant ovary cancer cell line and might be involved in the apoptotic response of cells under genotoxic stress caused by cisplatin [9]. CLPTM1L has been reported in the cellular response to genotoxic stress and cisplatin resistance [9]. In addition, over-expression of CLPTM1L has been observed in lung tumors [22]. In this study, we found that the expression of CLPTM1L was higher in the NPC tissues compared to the non-cancer nasopharyngeal tissues, suggesting that the CLPTM1Lhas been implicated in NPC carcinogenesis.

Chromosome 5p15.33 is one of the strongest and most consistent association signals for cancer risk [23]. Both GWAS and candidate gene association studies have identified common variants in 5p15.33 that reproducibly associate with several types of cancer. In 2008, McKay et al. carried out a GWAS of lung cancer, and two independent polymorphisms, rs402710 and rs2736100, were detected [12]. Another GWAS of lung cancer identified another associated polymorphism: rs401681 [11]. Subsequently, Rafnar et al. found that the rs401681 polymorphism was associated with the risk of basal cell, lung, urinary bladder, prostate and cervical cancers and cutaneous melanoma, and rs2736098, a synonymous coding SNP in exon 2 of the *TERT* gene, was associated with the risk of basal cell, lung, urinary bladder, and prostate cancers [10, 24]. Additionally, a meta-analysis of two GWAS of glioma demonstrated that the rs2736100 polymorphism was associated with the risk of glioma [16]. Petersen et al. conducted a GWAS of pancreatic cancer, and rs401681 was one of the most significantly associated polymorphisms [14]. GWAS of a testicular germ cell tumor identified two independently associated polymorphisms in the *TERT*-*CLPTM1L* locus, rs4635969 and rs2736100 [15]. Association studies of candidate gene strategies also reported that SNPs in *TERT* were associated with serous ovarian, breast, and endometrial cancers [25–27]. With regard to NPC, Fachiroh et al. and Ko et al. have reported that certain polymorphisms in the *TERT* gene were associated with NPC risk in Thailand [28, 29]. In this study, we found that two independent SNPs, rs2735845 and rs401681, were significantly associated with the risk of NPC. Our results, together with those of the earlier studies, suggest that the *TERT*-*CLPTM1L* locus may be a susceptibility region for NPC.

The molecular mechanism by which the rs401681 and rs2735845 polymorphisms confer cancer risk is not well characterized. The rs2735845 polymorphism, which resides in the intergenic region, was never reported before. The rs401681 polymorphism, which resides in an LD block that contains the *CLPTM1L* gene and the 5′ end of the *TERT* gene, is one of the most extensively studied polymorphisms in the *TERT*-*CLPTM1L* locus. Rafnar et al. reported that the rs401681 polymorphism may lead to an increase in the gradual shortening of telomeres over time, but the effect may only become apparent after a certain age [10]. In this study, we did not find any association between rs401681 genotypes and *TERT* and *CLPTM1L* mRNA levels. We found that the *CLPTM1L* mRNA levels transcribed by the rs2735845 *G* allele were higher than those transcribed by the CC genotype in fMSCs. However, the difference in the induction of the reporter gene expression between the rs2735845 two alleles is not statistically significant (data not shown). Further studies are warranted to elucidate the molecular mechanism of these two polymorphisms.

When reviewing the results of this study, several potential limits should be kept in mind. First, in this hospital-based study, our patients were enrolled from hospitals, and the controls were selected from the community population; thus, inherent selection bias cannot be completely excluded. However, through further adjustment and stratification in the data analyses, the potential confounding effect might have been minimized. Second, a tag SNP approach has been shown to improve the power of the study for common genetic variants; however, the modest effects from rare genetic variants may remain undetected. Deep re-sequencing of the *TERT*-*CLPTM1L* locus may provide further help to uncover additional associated variants and to facilitate selection of potential causal variants for further functional studies.

In conclusion, our study shows an association between the genetic variations in the *TERT-CLPTM1L* locus and the risk of occurrence of NPC in two Chinese subpopulations. These findings may provide support for the importance of the *TERT-CLPTM1L* locus in the pathogenesis of NPC. If confirmed by other studies, knowledge of genetic factors contributing to the pathogenesis of the NPC as presented here may have implications for the screening and treatment of this disorder.

## MATERIALS AND METHODS

### Study population

This study consisted of two populations of patients with NPC and controls residing in Guangxi and Guangdong provinces, both of which are located in southern China (see Supplementary Materials, Table S2). The Guangxi population, which was used for the 1st stage scanning analysis, consisted of 855 patients with NPC and 1036 controls. All subjects were unrelated ethnic Han Chinese and residents in Nanning city and the surrounding regions. The diagnosis of patients, the inclusion and exclusion criteria for patients and controls, and the definition of smokers and drinkers were described in detail in our previous studies [30]. Briefly, the patients with NPC were newly diagnosed and pathologically confirmed, and they were consecutively recruited at the Guangxi Cancer Hospital (Nanning, China) between September 2003 and January 2008. The response rate for case patients was 95%. Patients who received chemotherapy or radiotherapy before surgery or had another type of cancer were excluded from this study. The controls were randomly selected from a community cancer screening program for early detection of cancer conducted in the same regions during the same time period in which the NPC patients were collected. The response rate for control subjects was 91%. The selection criteria for the controls included no individual history of cancer. The Guangdong population, which was used for the 2nd stage replication analysis, consisted of 997 patients and 972 controls. The diagnosis of patients and the inclusion and exclusion criteria for patients and controls were described in detail previously [31]. Briefly, the patients with NPC were newly diagnosed and pathologically confirmed, and they were consecutively recruited from Sun Yat-sen University Cancer Center (SYSUCC, Guangzhou, China) between October 2005 and October 2007. Only the patients who were self-reported as Guangdong Chinese and who lived in Guangdong province were recruited. During the same period, healthy controls were recruited from physical examination centers of several large comprehensive hospitals in the local communities of Guangdong. The selection criteria for the controls included no individual history of cancer. The response rate for cases and control was 90%. For both populations, tumor staging was performed according to the tumor-node-metastasis (TNM) classification of the 1997 American Joint Committee on Cancer (AJCC) system. All TNM classifications were determined by senior pathologists of the hospital on the basis of the postoperative histopathologic examination.

Of the patients with NPC in the Guangxi population, 41 patients who had undergone resection before receiving any further treatment at Guangxi Cancer Hospital were selected, and primary NPC biopsies were collected from them (see Supplementary Materials, Table S3). All 41 tumor tissues were poorly differentiated squamous cell carcinoma (SCC). Histological non-cancerous nasopharyngeal epithelium tissues were collected from 13 of the 1036 control subjects in the Guangxi population (see Supplementary Materials, Table S3). All of the tissues were fixed in paraformaldehyde, embedded in paraffin wax, and prepared for IHC staining.

For *TERT* and *CLPTM1L* expression assays, human umbilical cord (UC) was obtained from a group of 12 normal full-term-delivery women. At recruitment, informed consent was obtained from all participants, and personal information about demographic factors and medical history and tobacco and alcohol use was collected via structured questionnaire. This study was performed with the approval of the Ethical Committee of Beijing Institute of Radiation Medicine and conducted according to the principles expressed in the Declaration of Helsinki.

### Selection of SNPs

Using the tagger program implemented in Haploview 4.1 [32], tag SNPs across the 115-kb of *TERT-CLPTM1L* locus on 5p15.33 (chr5:1,301,873–1,417,242) were selected on the basis of linkage disequilibrium (LD) patterns observed in the population of Han Chinese in Beijing, China (CHB), and genotyped as part of the International HapMap Project (data Rel 24/phase II, http://www.hapmap.org) (see Supplementary Materials, Figure S1). Two well-studied functional SNPs in the *TERT* gene (rs2853669 and rs2735940) and six SNPs (rs2736100, rs2736098, rs4975616, rs402710, rs401681, rs31489) reported in the GWAS studied were directly included as tag SNPs using the forced inclusion option [10–12, 33, 34]. In addition, 18 additional tag SNPs (rs3803309, rs4983540, rs3809454, rs3803304, rs2494738, rs2494743, rs2494750, rs4983559, rs2498806, rs10139525, rs8010024 and rs2582519) were selected with a pairwise *r*^2^ of > 0.80 and a minor allele frequency of ≥ 0.01 on the basis of HapMap CHB data. Overall, 26 tag SNPs in the *TERT*-*CLPTM1L* locus were selected for further genotyping (see Supplementary Materials, Table S4).

### Genotype analysis

In the stage I association study, 15 SNPs were genotyped using the MassArray System (Sequenom) with primers (see Supplementary Materials, Table S6) that were described previously [35]. All 15 SNPs had genotyping call rates of 95% or better. Nine SNPs were genotyped using the SNPstream ultra-high throughput genotyping system (Beckman Coulter, Fullerton, CA) according to the manufacturer's instructions, as described previously [36]. The primer sequences are listed in Table S6 in Supplementary Materials 1. The s2736118 polymorphism was eliminated because of poor genotyping reliability. The remaining eight SNPs had genotyping call rates of 95% or better. The rs2736100 polymorphism was genotyped by TaqMan MGB assay (Applied Biosystems, USA) according to the manufacturer's protocol, as described previously [30]. The primer and TaqMan probes used for the rs2736100 polymorphism were as follows: 5′-AGACGGGGAACAAAGGAGGA-3′ (forward primer), 5′-AGTTCTATCTCAGGCATCTTGACACC-3′ (reverse primer), 5′-FAM-CAAAGCTAAAGAAACAC-MGB-3′, 5′-HEX-CAAAGCTACAGAAACA-MGB-3′. The rs2853669 polymorphism was genotyped using polymerase chain reaction-based restriction fragment length polymorphism (PCR-RFLP) analysis. The primers and the restriction enzymes used are listed in Table S7 in Supplementary Materials 1. The PCR conditions were identical to those used for the SNP discovery described previously [37].

In the stage II association study, four SNPs (rs2736098, rs2735845, rs401710, and rs401681) were further genotyped in the Guangdong population. The genotyping data of the rs401710 and rs401681 polymorphisms were collected from Human610-Quad data published in Bei et al.'s study [31]. The rs2735845 and rs2736098 polymorphisms were genotyped by PCR-RFLP analysis (Table S6 in Supplementary Materials). The PCR conditions were identical to those for the rs2853669 polymorphism.

Genotyping was performed in a blind manner such that the performers did not know the subjects' patient or control status. For quality control, a 15% masked, random sample of patients and controls was tested twice by different people, and all results exhibited 100% concordance. We also designed PCR-RFLP assays to validate two random SNPs to compare with the results of the Sequneom and SNPstream methods, respectively, in 5% of the patient/control samples; the reproducibility was 98.8%. In addition, genotypes identified by PCR-RFLP were confirmed by DNA sequencing.

### Isolation and expansion of fMSCs from UC

UC (*n* = 12) was obtained from normal full-term-delivery women. Briefly, after disinfection with 75% ethanol, the fresh UC was transferred to PBS where excess blood on the surface was removed. After removal of blood vessels, 2 cm of UC was sliced into very small pieces followed by treatment with 0.1% collagenase II (Gibco, USA), containing antibiotic solution (100 U/mL penicillin and 100 μg/mL streptomycin; CSPC, China) in Dulbecco's Modified Eagle Medium/F12 (DMEM/F12) (Gibco) overnight at 37°C. The cells were cultured in DMEM/F12 supplemented with 10% fetal bovine serum (Hyclone, China) in 5% CO_2_ in a 37°C incubator for 3–4 days to allow cells to adhere. The adherent cells formed colonies and grew rapidly, exhibiting spindle-shaped morphology. The medium was replaced every three days. When the cells reached to 60–80% confluence, they were harvested with trypsin-EDTA solution (Neuronbc, China) and subcultured at a density of 3–6×10^3^ cells/cm^2^ [38]. Amplification of pluripotent stem cell markers, such as Oct-4 and Rex-1, was used to demonstrate primitive properties of the cultured cells, as described previously [39].

### Real-time analysis of *TERT* and *CLPTM1L* messenger RNA (mRNA) expression

Total RNA was extracted from 12 fetal MSCs using Trizol reagent (Invitrogen, USA) according to the manufacturer's protocol. Reverse transcription was performed using a reverse transcriptase system (Promega, USA) according to the manufacturer's instructions. Quantitative real-time PCR for *CLPTM1L* and full-length *TERT*, with *GAPDH* as an internal reference gene, was performed using the Bio-Rad IQ5 (Bio-Rad, USA) based on the SYBR-Green method. The primers used for *TERT* were 5′-TGTACTTTGTCAAGGTGGATGTG-3′ and 5′-GTAAGGCTGGAGGTCTGTCAA-3′ [40], those used for *CLPTM1L* were 5′-TAAGAGCTGGTACTCCTGGT-3′ and 5′-GTCATCAATGAAGGTGTTGA-3′, and those used for *GAPDH* were 5′-CATGAGAAGTATGACAACAGCCT-3′ and 5′-ACTCCTTCCACGATACCAAAGT-3′. All reactions performed in a 15-μL reaction volume containing 7.5 μL of 2X SYBR Green Master mix (Bio-Rad, USA), 1 μL of each primer pair (6 μmol/L), and 1 μL of cDNA templates. Cycling conditions were 95°C for 2 minutes, followed by 40 cycles at 95°C for 10 seconds, 54°C for 15 seconds, and 72°C for 15 seconds. The data were analyzed using the 2^−ΔΔCt^ method [41]. All analyses were performed in a blinded fashion with the laboratory persons unaware of the genotyping data.

### IHC staining analysis

NPC tissues (*n* = 41) and non-tumor nasopharyngeal tissues (*n* = 13), which had been fixed with paraformaldehyde and embedded in paraffin wax, were analyzed for protein expression of TERT and CLPTM1L. The IHC assay was performed as previously described [42]. Briefly, two slides of each biopsy were stained with hematoxylin-eosin for routine histological evaluation. The slides were washed in xylene to remove the paraffin and then rehydrated through serial dilutions of alcohol, followed by washings with a solution of PBS (pH 7.2). All subsequent washes were buffered via the same protocol. The slides were then incubated with 3% H_2_O_2_ for 10 min to reduce non-specific staining. The treated sections were then placed in a citrate buffer (pH 6.0) and heated in a pressure cooker for two minutes. The samples were then incubated with a polyclonal anti-human CLPTM1L antibody (Sigma Aldrich, HPA014791) or a monoclonal rabbit anti-human TERT antibody (Lifespan, LS-C49516) overnight at 4°C. The conventional streptavidin peroxidase method (MaxVision^™^ HRP-Polymer IHC Kit; Maxim Co., China) was performed for signal development, and the cells were counter-stained with hematoxylin. Negative controls and positive controls were prepared at the same time. The slides were mounted with gum for examination and capture by the Olympus BX51 microscopic/Digital Camera System for study comparison. IHC signals were scored as previously described [42]. Slides were scored by two pathologists (Wu JH and Wang AC) who did not have knowledge of the genotype results of rs2735845 and rs401681 polymorphisms or of the patient outcomes.

### Statistical analysis

Genotype and allele frequencies for polymorphisms were determined by gene counting. The fitness according to the Hardy-Weinberg equilibrium was tested using the *χ*^2^ test. Associations between polymorphisms and risk of NPC were estimated by use of logistic regression analyses. Odds ratios (ORs) and 95% confidence intervals (CIs) were used to measure the strength of association. To correct for multiple testing, we used spectral decomposition (SpD) of matrices of LD between SNPs, which adjusts for multiple testing while taking into account LD among the tested SNPs [43]. This method showed that *P* values of 0.0029 and below can be considered statistically significant after correction for multiple testing. These analyses were performed using SPSS software (version 17.0, SPSS Inc., Chicago, IL, USA). The pairwise LD calculation (Lewontin's *D* and *r*^2^) and haplotype block construction were performed using the program Haploview 4.2 [32]. The haplotype analysis was performed using PLINK software (version 1.07) (http://pngu.mgh.harvard.edu/purcell/plink/) [44].

## SUPPLEMENTARY MATERIAL TABLES AND FIGURE


