# Supplementary material for: Common variations in TERT-CLPTM1L locus are reproducibly associated with the risk of nasopharyngeal carcinoma in Chinese populations
Source: Oncotarget. 2015 Nov 26;7(1):759–70. doi: 10.18632/oncotarget.6397 (PMC4808031; doi:10.18632/oncotarget.6397)
Supplement: Supplementary file 1 [file oncotarget-07-0759-s001.pdf]

Common variations in TERT-CLPTM1L locus are reproducibly associated with the risk of nasopharyngeal carcinoma in Chinese populations

Supplementary Materials

Supplementary Table S1: Correlation between protein expression levels of TERT and CLPTM1L and rs2735845 and rs401681 genotypes in nasopharyngeal carcinoma tissues and non-cancer nasopharyngeal tissues by immunohistochemistry

Protein	Tissue	Genotypes	Expression levels[a], *n*			*P* value[b]	Genotype	Expression levels[a], *n*			*P* value[b]	*P* value[c]
			Negative	Low	High	CC vs. CG + GG		Negative	Low	High	CC vs. CT + TT	Tumor vs. non-tumor tissues
TERT		rs2735845					rs401681					
						0.774					0.386	4.78×10^{-7}
	Tumor	CC	3	3	6		CC	2	4	11		
		CG	4	6	12		CT	4	7	8		
		GG	0	1	3		TT	1	0	1		
	Non-NPC tissues					NA					NA	
		CC	4	0	0		CC	9	0	0		
		CG	6	0	0		CT	3	0	0		
		GG	2	0	0		TT	0	0	0		
CLPTM1L						0.415					0.347	4.70×10^{-12}
	Tumor	CC	0	10	2		CC	1	13	4		
		CG	0	12	0		CT	0	11	8		
		GG	0	5	0		TT	0	2	0	NA	
	Non-NPC tissues					NA						
		CC	5	0	0		CC	11	0	0		
		CG	6	0	0		CT	3	0	0		
		GG	3	0	0		TT	0	0	0		

Abbreviations: NA, not applicable.

[a]Expression levels were classified into three groups (negative, low, and high expression) with scores of the immunohistochemistry signals.

[b]The differences of the protein levels between the genotypes were assessed by χ^2 test.

[c]The differences of the protein levels between the tumors and non-cancer nasopharyngeal tissues were assessed by a Wilcox test.

Supplementary Table S2: Selected characteristics of patients and controls of the Guangxi and Guangdong population

Characteristics	Guangxi population, *n* (%)			Guangdong population, *n* (%)		
	Cases (*n* = 855)	Controls (*n* = 1036)	*P* value[a]	Cases (*n* = 997)	Controls (*n* = 972)	*P* value[a]
Age, year						
Mean ± SD	46.37±11.90	44.7±12.1	0.001	47.36±11.10	47.40±11.59	0.93
< mean	391 (45.7)	520 (50.2)	0.05	414 (41.5)	394 (40.5)	0.66
Men	616 (72.0)	749 (72.3)	0.9	728 (73.0)	709 (72.9)	0.97
Smoking status, *n* (%)						
Smoker	257 (30.1)	317 (30.6)	0.80	546 (54.8)	506 (52.1)	0.15
Non-smoker	598 (69.9)	719 (69.4)		441 (44.2)	466 (47.9)	
Unknown	0	0		10 (1.0)	0	
Smoking level, pack-years						
Mean ± SD	22.9±12.9	24.3±18.4	0.26	14.22±18.54	11.69±17.05	0.10
< mean	106 (41.2)	128 (40.4)	0.83	228 (42.2)	276 (55.1)	< 0.001
Drinking status, *n* (%)						
Drinker	256 (29.9)	302 (29.2)	0.71	178 (17.9)	150 (15.4)	0.11
Non-drinker	599 (70.1)	734 (70.8)		802 (80.4)	822 (84.6)	
Unknown	0	0		17 (1.7)	0	
First-family history			< 0.001			< 0.001
Negative	797 (93.2)	1006 (97.1)		821 (82.3)	933 (96.0)	
Positive	58 (6.8)	30 (2.9)		168 (16.9)	26 (2.7)	
Unknown	0	0		8 (0.8)	13 (1.3)	
Nationality, *n* (%)			< 0.001			
Han	628 (73.5)	904 (87.3)				
Non-Han[b]	227 (26.5)	132 (12.7)				
Histological type						
Poorly differentiated squamous cell carcinoma	829 (97.0)					
Others[c]	26 (3.0)					
Clinical stage						
I	41 (4.8)			36 (3.6)		
II	395 (46.2)			149 (14.9)		
III	259 (30.3)			503 (50.5)		
IV	160 (18.7)			253 (25.4)		
Unknown	0			56 (5.6)		

Local tumor invasion (T classification)						
T1	170 (19.9)			56 (5.6)		
T2	424 (49.6)			229 (23.0)		
T3	174 (20.4)			482 (48.3)		
T4	87 (10.1)			194 (19.5)		
Unknown	0			36 (3.6)		
Lymph node involvement (N classification)						
N0	178 (20.8)			235 (23.6)		
N1	414 (48.4)			384 (38.5)		
N2	185 (21.6)			274 (27.5)		
N3	78 (9.2)			69 (6.9)		
Unknown	0			35 (3.5)		
Distance metastasis (M classification)						
M0	835 (97.7)			935 (93.8)		
M1	20 (2.3)			25 (2.5)		
Unknown	0			37 (3.7)		

Abbreviations: SD, standard deviation.

[a] χ^2 test for categorical variables and unpaired t test for continuous variables.

[b] In the patients of Guangxi population, non-Han includes Zhuang (*n* = 211), Dong (*n* = 1), Hui (*n* = 1), Miao (*n* = 1), Mulao (*n* = 3) and Yao (*n* = 10) nationality; in the controls, non-Han includes Zhuang (*n* = 132) nationality.

[c] Other histological types include vesicular nucleus cell carcinoma (*n* = 14), poorly differentiated adenocarcinoma (*n* = 4), and moderate differentiated squamous cell carcinoma (*n* = 5); and undifferentiated cancer (*n* = 3).

Supplementary Table S3: Selected characteristics of patients with nasopharyngeal carcinoma and controls for the immunohistochemical staining in nasopharyngeal carcinoma tissues and non-tumor nasopharyngeal tissues

Characteristics	Cases (*n* = 41)	Controls (*n* = 13)
Age, years		
Mean (SD)	44.3 (11.8)	36.8 (12.5)
Men, *n* (%)	23 (56.1)	10 (76.9)
Smoker, *n* (%)	13 (31.7)	1 (92.3)
Smoking level, pack-years		
Mean (SD)	7.65 (13.0)	3.18 (11.1)
Drinker, *n* (%)	7 (17.1)	0 (0)
Nationality, *n* (%)		
Han	36 (87.8)	13 (100)
Non-Han[a]	5 (12.2)	0 (0)
Histological type, *n* (%)[b]		
Poorly differentiated squamous cell carcinoma	41 (100)	
Others	0 (0)	

Clinical stage, *n* (%)		
I	0 (0)	
II	34 (82.9)	
III	4 (9.8)	
IV	3 (7.3)	
Local tumor invasion (T classification), *n* (%)		
T1	1 (2.4)	
T2	27 (65.9)	
T3	10 (24.4)	
T4	3 (7.3)	
Lymph node involvement (N classification), *n* (%)		
N0	3 (7.31)	
N1	27 (65.9)	
N2	11 (26.7)	
N3	0 (0)	
Distance metastasis (M classification), *n* (%)		
M0	40 (97.6)	
M1	1 (2.4)	

Abbreviations: SD, standard deviation.
[a]In cases, all the non-Han is Zhuang (n = 5) nationality.
[b]The histological type of all tumor tissues was poorly differentiated squamous cell carcinoma. The histological type of all tissues in the controls was chronic inflammation.

Supplementary Table S4: 26 tag SNPs selected to be genotyped in Guangxi population

No.	Chromosome position	SNPs	Genes	Gene position	Type of SNP
1	1238873	rs6554691		3′-UTR	Tag SNP
2	1239594	rs10078761		3′-UTR	Tag SNP
3	1242950	rs2853691	*TERT*	3′-UTR	Tag SNP
4	1250195	rs2736118	*TERT*	Intron 12	Tag SNP
5	1256310	rs2075786	*TERT*	Intron 10	Tag SNP
6	1257356	rs4246742	*TERT*	Intron 9	Tag SNP
7	1265528	rs4975605	*TERT*	Intron 6	Tag SNP
8	1269790	rs10069690	*TERT*	Intron 4	Tag SNP
9	1276516	rs2736100	*TERT*	Intron 2	GWAS reported [1, 2]
10	1278547	rs2853676	*TERT*	Intron 2	Tag SNP
11	1284086	rs2736098	*TERT*	Exon 2	GWAS reported [3]
12	1285349	rs2853669	*TERT*	5′-UTR	Functional SNP [4]
13	1286486	rs2735940	*TERT*	5′-UTR	Functional SNP [5]
14	1290025	rs2853668		Intergenic	Tag SNP
15	1290584	rs2735845		Intergenic	Tag SNP
16	1305660	rs4975616		Intergenic	GWAS reported [1, 2]
17	1307102	rs6554759	*CLPTM1L*	3′-UTR	Tag SNP

18	1309680	rs451360	*CLPTM1L*	Intron 16	Tag SNP
19	1310722	rs402710	*CLPTM1L*	Intron 16	GWAS reported [1]
20	1311873	rs10073340	*CLPTM1L*	Intron 14	Tag SNP
21	1312087	rs401681	*CLPTM1L*	Intron 13	GWAS reported [1, 2, 3]
22	1332714	rs31489	*CLPTM1L*	Intron 2	GWAS reported [1, 2]
23	1349938	rs27064		5′-UTR	Tag SNP
24	1350125	rs27063		5′-UTR	Tag SNP
25	1352793	rs27061		5′-UTR	Tag SNP
26	1354242	rs2292024		5′-UTR	Tag SNP

Abbreviations: 5′-untranslated region, 5′-UTR; genome-wide association studies, GWAS.

Table S5: Primers used in Sequenom platform

No.	SNPs	Gene	Primers	Sequences
1	rs10078761		1st-PCR	5′-ACGTTGGATGATGAGTCTCCCGTCCACACT-3′
			2nd-PCR	5′-ACGTTGGATGTCCTCTGACACAGCACGAAG-3′
			Extension	5′-GCCACCTGGAGTGGTA-3′
2	rs2736118	*TERT*	1st-PCR	5′-ACGTTGGATGTCAGAGCCTCAGCCCACCAA-3′
			2nd-PCR	5′-ACGTTGGATGCTAGGTCCTCAGCACCAGT-3′
			Extension	5′-ccctcGCCACTCCTTACAGG-3′
3	rs4246742	*TERT*	1st-PCR	5′-ACGTTGGATGAGGCCACACAGCCATTTCTC-3′
			2nd-PCR	5′-ACGTTGGATGTGCTATAATTGGAGCTAGAG-3′
			Extension	5′-caaacGATGCACAGGAAAAACCCGGAG-3′
4	rs10069690	*TERT*	1st-PCR	5′-ACGTTGGATGACCCCGTCATCTGAGGAGA-3′
			2nd-PCR	5′-ACGTTGGATGATGTGTGTTGCACACGGGAT-3′
			Extension	5′-GGGATCCTCATGCCA-3′
5	rs2853676	*TERT*	1st-PCR	5′-ACGTTGGATGTGTCTCCTGCTCTGAGACC-3′
			2nd-PCR	5′-ACGTTGGATGACTAAGACCCAAGAGGGAAG-3′
			Extension	5′-CCGCCAGCACCACGCA-3′
6	rs2736098	*TERT*	1st-PCR	5′-ACGTTGGATGTGGTGGCCGCGATGTGGAT-3′
			2nd-PCR	5′-ACGTTGGATGTCTCTGGCACGCGCCACTC-3′
			Extension	5′-CCGCCAGCACCACGC-3′
7	rs2735940	*TERT*	1st-PCR	5′-ACGTTGGATGAGCCTCGTCTTGTAAATAC-3′
			2nd-PCR	5′-ACGTTGGATGAGGCTTAGGGATCACTAAGG-3′
			Extension	5′-tGGATTTCTAGAAGAGCGACC-3′
8	rs2735845	*TERT*	1st-PCR	5′-ACGTTGGATGGACCTTGGCAAACATAGCTC-3′
			2nd-PCR	5′-ACGTTGGATGCTTAAATTTCATCAAATAAC-3′
			Extension	5′-tcAATTTCATCAAATAACATTCAGGA-3′
9	rs402710	*CLPTM1L*	1st-PCR	5′-ACGTTGGATGACGCCGCTGAGACGGAGCAA-3′
			2nd-PCR	5′-ACGTTGGATGTGGTGTTTCTGGTCTACCTG-3′
			Extension	5′-gaaaAGCGGTGGTGAGTGC-3′

10	rs401681	*CLPTM1L*	1st-PCR	5′-ACGTTGGATGATGCATAGTGGGCAGAAAAC-3′
			2nd-PCR	5′-ACGTTGGATGGCCAGAAAGCTGCTTCACAC-3′
			Extension	5′-tttTGCTTCACACCATGAT-3′
11	rs31489	*CLPTM1L*	1st-PCR	5′-ACGTTGGATGTACACTTTCAGCCTGGTGAC-3′
			2nd-PCR	5′-ACGTTGGATGTACGGTTACATGAGTTCTTC-3′
			Extension	5′-aagGAGTTCTTCTTCCTCTTTAAAAGT-3′
12	rs27064		1st-PCR	5′-ACGTTGGATGTCTCCGTGTTTTGCTCTGAC-3′
			2nd-PCR	5′-ACGTTGGATGTCTGCCGTCTTAGCTGGAAA-3′
			Extension	5′-CTGCAAGTTTTTCTCAAGATGTAG-3′
13	rs27063		1st-PCR	5′-ACGTTGGATGCAGTGTGAGACATCATAGCC-3′
			2nd-PCR	5′-ACGTTGGATGGAGCAATTCACAGATGAGCG-3′
			Extension	5′-acaaAGATGAGCGCGGGCA-3′
14	rs27061		1st-PCR	5′-ACGTTGGATGGCCACGCACTTAAGAATAGG-3′
			2nd-PCR	5′-ACGTTGGATGTGTCGTGAATCGAGGAACAG-3′
			Extension	5′-cccTCCACAAAAAAAGCGAG-3′
15	rs2292024		1st-PCR	5′-ACGTTGGATGAGAGAACCTGGCATCCTTTC-3′
			2nd-PCR	5′-ACGTTGGATGAAGAACCCTGGGAGGAAGAC-3′
			Extension	5′-GCTGGGTACCTGAAAT-3′

Table S6: Primers used in SNPstream platform

No.	Genes	SNPs	Amplicon size (bp)	Primers	Sequences
1		rs6554691	95	Forward	5′-TTGTTCAGGGACAGGGGC-3′
				Reverse	5′-TTCTGCTCTCGCCGCCTG-3′
				Extension	5′-GGATGGCGTTCCGTCCTATTTGTCTCCCCCTC CCTCGATGGTGC-3′
2	*TERT*	rs2853691	136	Forward	5′-TGGAAGACATAACAGTAAGTCCAG-3′
				Reverse	5′-ATCACCCCTGCCACCCCA-3′
				Extension	5′-CAGCACTATTACCATCACGTGGCGGGGATGATG GAGGGCCTGGCC-3′
3	*TERT*	rs10069690	96	Forward	5′-TTTGAAACGGGTTCCTGG-3′
				Reverse	5′-ACCCCGTCATCTGAGGAG-3′
				Extension	5′-AGATAGAGTCGATGCCAGCTTGTTGCACACG GGATCCTCATGCCA-3′
4	*TERT*	rs2853669	102	Forward	5′-CGGGCTCCCAGTGGATTC-3′
				Reverse	5′-TGGAAGGTGAAGGGGCAG-3′
				Extension	5′-CTCAGACTACGAATCCACGTGGGCACAGACGC CCAGGACCGCGCT-3′
5		rs2853668	151	Forward	5′-GTTCCTTCCATCATTATTCATCTT-3′
				Reverse	5′-ACTCTTAATATATCAGGGGTGTGG-3′
				Extension	5′-AGCCGAACTACCACTGAGTACTTCGGGTGTGA CAAGCCATGACAA-3′

6	*CLPTM1L*	rs6554759	132	Forward	5′-ATTCCTCTCAAATCGAGTGAAGT-3′
				Reverse	5′-TGAGAACCAAATCCAGATGC-3′
				Extension	5′-AGCGATCTGCGAGACCGTATAATTATAAAAGATTTATCTGATCCA-3′
7	*CLPTM1L*	rs451360	119	Forward	5′-TACTAGTGCAGGAGAGGGATCC-3′
				Reverse	5′-TTGAGTGTGGAGACAGGGC-3′
				Extension	5′-CAGCACTATTACCATCACGTACGGGAGCACTGCGCTCCCCAAGGG-3′
8	*CLPTM1L*	rs402710	101	Forward	5′-ATTCCGTTCAGCAGCAGC-3′
				Reverse	5′-GACGTGGTGTTTCTGGTCTAC-3′
				Extension	5′-AGAGCGAGTGACGCATACTAtacctgtaccagcggtggtgagtgc-3′
9	*CLPTM1L*	rs10073340	97	Forward	5′-TGGGCAGCACAGGAGACG-3′
				Reverse	5′-TTTGGTTTCCTCTTCATGCT-3′
				Extension	5′-CAGAACATCCTCAGAAGCAAAGCTCTTTGTGAACTACAAGGTAAG-3′

Table S7: Primers used in polymerase chain reaction/restriction fragment length polymorphism

SNPs	Primers	Sequences	Amplicon size (bp)	Restriction enzyme	Digest size (bp)
rs2736098	Forward	5′-GAGTGACCGTGGTTTCTGTGTG-3′	305	*Bsp120*I	GG: 180, 125
	Reverse	5′-CCAGAAAGATGGTCTCCACGAG-3′			AA: 305
rs2853669	Forward	5′-ATTCGCGGGCACAGACGCCCAGGACCGAGCT-3′	230	*Sac*I	TT: 230, 31
	Reverse	5′-CAGaGCTGCCTGAAACTC-3′			CC: 199
rs2735845	Forward	5′-CTGATCGGGACAGAGTGACC-3′	352	*Pst*I	CC: 259, 93
	Reverse	5′-TTAGGGCGAAAAATCCCTCT-3′			GG:352

Table S8: Associations between 22 tag SNPs on chromosome 5p15.33 and tumor (T), node (N), metastasis (M) stage of NPC in the Guangxi population

SNP	Gene	Genotypes	Case n (%) $T = 1$	Case n (%) $T = 2, 3, 4$	OR (95% CI)[a]	P[a]	Case n (%) $N = 0$	Case n (%) $N = 1, 2, 3$	OR (95% CI)[a]	P[a]	Case n (%) $M = 0$	Case n (%) $M = 1$	OR (95% CI)[a]	P[a]
rs6554691		A/A	107 (65.6)	405 (64.9)	1.03 (0.75–1.42)	0.84	110 (66.7)	402 (64.6)	1.07 (0.78–1.46)	0.67	500 (65.1)	12 (63.2)	1.17 (0.53–2.60)	0.70
		T/A	50 (30.7)	195 (31.2)			49 (29.7)	196 (31.5)			239 (31.1)	6 (31.6)		
		T/T	6 (3.7)	24 (3.8)			6 (3.6)	24 (3.9)			29 (3.8)	1 (5.3)		
rs10078761		T/T	163 (96.5)	601 (94)	1.72 (0.72–4.12)	0.20	159 (93.5)	605 (94.8)	0.81 (0.41–1.62)	0.56	746 (94.7)	18 (90)	2.02 (0.46–8.87)	0.39
		T/A	6 (3.5)	37 (5.8)			11 (6.5)	32 (5)			41 (5.2)	2 (10)		
		A/A	0 (0)	1 (0.2)			0 (0)	1 (0.2)			1 (0.1)	0 (0)		
rs2075786	*TERT*	A/A	117 (71.3)	428 (66.4)	1.28 (0.92–1.78)	0.14	121 (70.8)	424 (66.5)	1.24 (0.90–1.71)	0.18	534 (67.6)	11 (57.9)	1.22 (0.55–2.71)	0.62
		G/A	43 (26.2)	189 (29.3)			46 (26.9)	186 (29.1)			224 (28.4)	8 (42.1)		
		G/G	4 (2.4)	28 (4.3)			4 (2.3)	28 (4.4)			32 (4)	0 (0)		
rs4246742	*TERT*	T/T	46 (28.1)	201 (31.4)	0.91 (0.71–1.16)	0.44	57 (33.7)	190 (29.9)	1.18 (0.93–1.51)	0.17	243 (31)	4 (20)	1.01 (0.53–1.90)	0.98
		T/A	81 (49.4)	311 (48.5)			83 (49.1)	309 (48.6)			378 (48.1)	14 (70)		
		A/A	37 (22.6)	129 (20.1)			29 (17.2)	137 (21.5)			164 (20.9)	2 (10)		
rs4975605	*TERT*	C/C	116 (71.6)	497 (75.8)	0.84 (0.60–1.16)	0.29	131 (75.3)	482 (74.8)	1.02 (0.74–1.42)	0.9	598 (74.8)	15 (79)	0.91 (0.36–2.33)	0.85
		C/A	41 (25.3)	137 (20.9)			37 (21.3)	141 (21.9)			175 (21.9)	3 (15.8)		
		A/A	5 (3.1)	22 (3.4)			6 (3.5)	21 (3.3)			26 (3.2)	1 (5.3)		
rs10069690	*TERT*	C/C	130 (77.8)	455 (71.2)	1.37 (0.94–2.01)	0.096	124 (72.9)	461 (72.5)	1.02 (0.72–1.45)	0.91	570 (72.4)	15 (79)	0.70 (0.24–2.07)	0.51
		T/C	35 (21)	174 (27.2)			43 (25.3)	166 (26.1)			205 (26.1)	4 (21.1)		
		T/T	2 (1.2)	10 (1.6)			3 (1.8)	9 (1.4)			12 (1.5)	0 (0)		
rs2736100	*TERT*	T/T	50 (30.9)	192 (30.8)	0.98 (0.76–1.27)	0.90	45 (27.6)	197 (31.7)	0.88 (0.68–1.13)	0.31	234 (30.5)	8 (44.4)	0.74 (0.36–1.50)	0.40
		T/G	79 (48.8)	314 (50.4)			84 (51.5)	309 (49.7)			386 (50.3)	7 (38.9)		
		G/G	33 (20.4)	117 (18.8)			34 (20.9)	116 (18.6)			147 (19.2)	3 (16.7)		

rs2853676	*TERT*	C/C	118 (71.5)	477 (75.5)	0.89 (0.63–1.26)	0.52	125 (74)	470 (74.8)	0.93 (0.67–1.30)	0.68	582 (74.7)	13 (72.2)	1.26 (0.54–2.95)	0.61
		C/T	45 (27.3)	134 (21.2)			38 (22.5)	141 (22.4)			175 (22.5)	4 (22.2)		
		T/T	2 (1.2)	21 (3.3)			6 (3.5)	17 (2.7)			22 (2.8)	1 (5.6)		
rs2736098	*TERT*	C/C	55 (34.2)	177 (28.8)	1.23 (0.95–1.60)	0.12	44 (27.9)	188 (30.4)	0.83 (0.64–1.07)	0.16	225 (29.7)	7 (36.8)	0.93 (0.47–1.85)	0.83
		T/C	81 (50.3)	322 (52.4)			78 (49.4)	325 (52.6)			395 (52.2)	8 (42.1)		
		T/T	25 (15.5)	116 (18.9)			36 (22.8)	105 (17)			137 (18.1)	4 (21.1)		
rs2853669	*TERT*	T/T	64 (38.5)	232 (37.3)	1.09 (0.85–1.40)	0.50	60 (37.3)	236 (37.6)	0.90 (0.70–1.16)	0.41	290 (37.6)	6 (35.3)	1.04 (0.50–2.13)	0.92
		T/C	78 (47)	290 (46.6)			69 (42.9)	299 (47.7)			359 (46.6)	9 (52.9)		
		C/C	24 (14.5)	100 (16.1)			32 (19.9)	92 (14.7)			122 (15.8)	2 (11.8)		
rs2735940	*TERT*	A/A	33 (20.6)	189 (29.9)	0.81 (0.63–1.05)	0.11	60 (35.7)	162 (26)	1.32 (1.03–1.69)	0.025	218 (28.2)	4 (23.5)	1.07 (0.54–2.12)	0.85
		G/A	90 (56.2)	300 (47.5)			75 (44.6)	315 (50.6)			381 (49.2)	9 (52.9)		
		G/G	37 (23.1)	142 (22.5)			33 (19.6)	146 (23.4)			175 (22.6)	4 (23.5)		
rs2735845		C/C	64 (39.3)	210 (33.1)	1.15 (0.89–1.48)	0.28	55 (32.9)	219 (34.7)	0.88 (0.69–1.13)	0.31	264 (33.9)	10 (55.6)	0.44 (0.20–0.97)	0.030
		G/C	72 (44.2)	309 (48.7)			78 (46.7)	303 (48)			374 (48)	7 (38.9)		
		G/G	27 (16.6)	116 (18.3)			34 (20.4)	109 (17.3)			142 (18.2)	1 (5.6)		
rs4975616		A/A	99 (60.4)	384 (59.6)	1.00 (0.74–1.36)	0.98	108 (63.2)	375 (58.9)	1.19 (0.88–1.61)	0.26	472 (59.9)	11 (55)	1.39 (0.68–2.82)	0.37
		G/A	57 (34.8)	232 (36)			57 (33.3)	232 (36.4)			282 (35.8)	7 (35)		
		G/G	8 (4.9)	28 (4.3)			6 (3.5)	30 (4.7)			34 (4.3)	2 (10)		
rs6554759	*CLPTM1L*	A/A	141 (84.4)	551 (87.5)	0.76 (0.47–1.24)	0.28	143 (86.1)	549 (87)	0.96 (0.58–1.58)	0.87	676 (86.9)	16 (84.2)	1.32 (0.37–4.69)	0.68
		G/A	26 (15.6)	78 (12.4)			23 (13.9)	81 (12.8)			101 (13)	3 (15.8)		
		G/G	0 (0)	1 (0.2)			0 (0)	1 (0.2)			1 (0.1)	0 (0)		
rs451360	*CLPTM1L*	T/T	117 (70.9)	446 (68.8)	1.12 (0.80–1.57)	0.49	125 (75.3)	438 (67.7)	1.38 (0.97–1.95)	0.064	549 (69.1)	14 (73.7)	1.21 (0.53–2.77)	0.65
		T/G	44 (26.7)	182 (28.1)			37 (22.3)	189 (29.2)			223 (28.1)	3 (15.8)		
		G/G	4 (2.4)	20 (3.1)			4 (2.4)	20 (3.1)			22 (2.8)	2 (10.5)		

rs402710	*CLPTM1L*	G/G	77 (45.8)	339 (50.5)	0.80 (0.61–1.04)	0.10	87 (49.4)	329 (49.5)	1.05 (0.80–1.37)	0.72	408 (49.8)	8 (40)	1.34 (0.68–2.61)	0.40
		G/A	72 (42.9)	286 (42.6)			77 (43.8)	281 (42.3)			348 (42.4)	10 (50)		
		A/A	19 (11.3)	47 (7)			12 (6.8)	54 (8.1)			64 (7.8)	2 (10)		
rs401681	*CLPTM1L*	C/C	77 (46.1)	319 (49.8)	0.82 (0.63–1.07)	0.15	87 (51.2)	309 (48.4)	1.05 (0.80–1.37)	0.73	389 (49.2)	7 (38.9)	1.42 (0.70–2.85)	0.34
		C/T	71 (42.5)	278 (43.4)			68 (40)	281 (44)			340 (43)	9 (50)		
		T/T	19 (11.4)	44 (6.9)			15 (8.8)	48 (7.5)			61 (7.7)	2 (11.1)		
rs31489	*CLPTM1L*	C/C	98 (59.4)	385 (60.6)	0.96 (0.71–1.29)	0.77	106 (63.1)	377 (59.6)	1.20 (0.89–1.63)	0.23	470 (60.3)	13 (65)	1.05 (0.50–2.23)	0.89
		C/A	59 (35.8)	219 (34.5)			57 (33.9)	221 (35)			273 (35)	5 (25)		
		A/A	8 (4.8)	31 (4.9)			5 (3)	34 (5.4)			37 (4.7)	2 (10)		
rs27064		C/C	77 (47.2)	286 (44.8)	1.08 (0.83–1.40)	0.58	82 (47.4)	281 (44.7)	0.98 (0.76–1.26)	0.86	353 (45.1)	10 (52.6)	0.71 (0.34–1.49)	0.35
		C/T	69 (42.3)	279 (43.7)			66 (38.1)	282 (44.8)			340 (43.4)	8 (42.1)		
		T/T	17 (10.4)	74 (11.6)			25 (14.4)	66 (10.5)			90 (11.5)	1 (5.3)		
rs27063		G/G	43 (25.6)	165 (25.8)	0.94 (0.73–1.20)	0.62	45 (26.5)	163 (25.6)	1.07 (0.84–1.36)	0.60	204 (25.9)	4 (20)	1.52 (0.79–2.90)	0.20
		C/G	81 (48.2)	326 (50.9)			88 (51.8)	319 (50)			399 (50.6)	8 (40)		
		C/C	44 (26.2)	149 (23.3)			37 (21.8)	156 (24.4)			185 (23.5)	8 (40)		
rs27061		C/C	46 (27.5)	177 (27.6)	0.99 (0.77–1.26)	0.91	44 (26.2)	179 (28)	1.03 (0.81–1.31)	0.83	220 (27.9)	3 (15)	1.91 (0.99–3.67)	0.047
		C/T	79 (47.3)	314 (49)			88 (52.4)	305 (47.7)			385 (48.9)	8 (40)		
		T/T	42 (25.1)	150 (23.4)			36 (21.4)	156 (24.4)			183 (23.2)	9 (45)		
rs2292024		C/C	126 (76.8)	503 (76.7)	1.10 (0.77–1.58)	0.59	134 (78.4)	495 (76.3)	1.03 (0.73–1.45)	0.87	612 (76.5)	17 (85)	0.60 (0.19–1.91)	0.35
		G/C	36 (21.9)	132 (20.1)			31 (18.1)	137 (21.1)			165 (20.6)	3 (15)		
		G/G	2 (1.2)	21 (3.2)			6 (3.5)	17 (2.6)			23 (2.9)	0 (0)		

[a]ORs and *P* values were calculated under log-additive genetic models and adjusted for age, sex, smoke, drink, nationality and family history of cancer.

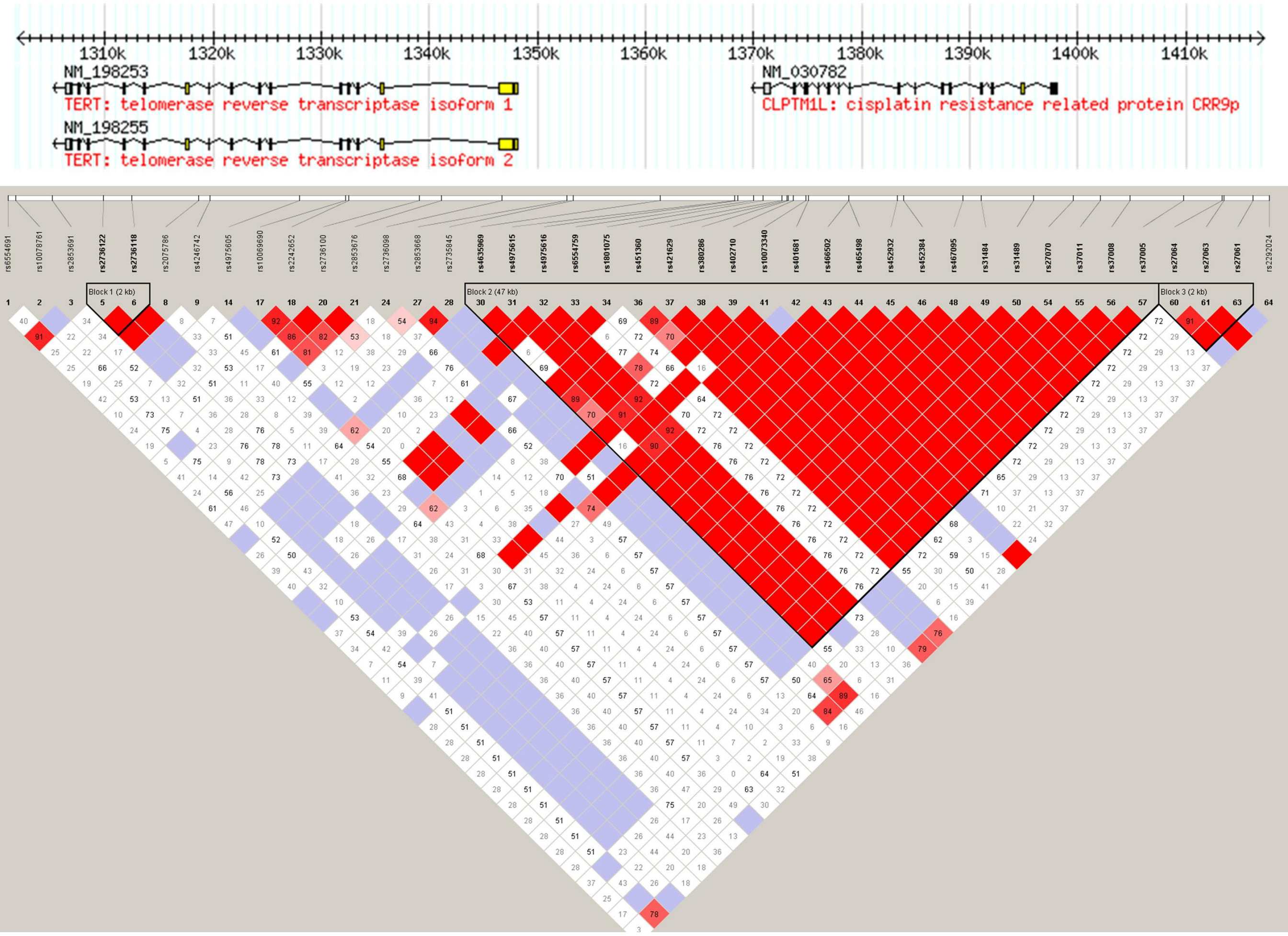

Supplementary Figure S1: Overview of linkage disequilibrium (LD) of the 115 kb-region of TERT-CLPTM1L. The LD structure around the TERT-CLPTM1L region of chromosome 5 (1,301,873 to 1,417,242) was derived from the genotyping data of SNPs in the CHB (Beijing Han Chinese) population from the HapMap database (http://www.hapmap.org). The value within each diamond represents the pairwise correation between tag SNPs (measured as *D*') defined by the upper left and the upper right sides of the diamond. The diamond without a number corresponds to *D*' = 1. Shading represents the magnitude and significance of pairwise LD, with a red-to-white gradient reflecting higher to lower LD values.

REFERENCES

1. McKay JD, Hung RJ, Gaborieau V, Boffetta P, Chabrier A, et al. Lung cancer susceptibility locus at 5p15.33. Nat Genet. 2008; 40: 1404–1406.
2. Wang, Y, P. Broderick, et al. Common 5p15.33 and 6p21.33 variants influence lung cancer risk. Nat Genet. 2008; 40: 1407–1409.
3. Rafnar, T, P. Sulem, et al. Sequence variants at the TERT-CLPTM1L locus associate with many cancer types. Nat Genet. 2009; 41: 221–227.
4. Hsu, C. P., N. Y. Hsu, et al. Ets2 binding site single nucleotide polymorphism at the hTERT gene promoter—effect on telomerase expression and telomere length maintenance in non-small cell lung cancer. Eur J Cancer. 2006; 42: 1466–1474.
5. Matsubara, Y, M. Murata, et al. Telomere length of normal leukocytes is affected by a functional polymorphism of hTERT. Biochem Biophys Res Commun. 2006; 341: 128–131.
6. Mushiroda T, Wattanapokayakit S, Takahashi A, Nukiwa T, Kudoh S, et al. A genome-wide association study identifies an association of a common variant in TERT with susceptibility to idiopathic pulmonary fibrosis. J Med Genet. 2008; 45: 654–656.
